# Editorial

**Published:** 1855-09

**Authors:** 


### THE MEDICAL EXAMINER.

### PHILADELPHIA, SEPTEMBER, 1855.

There is no class of invalids who require the comforts, conveniences, and even the luxuries of life more than those threatened with consumption; and yet it oftentimes has happened to those who have sought the equable temperature of Florida during the winter and spring months, to be so seriously inconvenienced from the want of proper accommodation, adapted to their condition, that the benefits they had hoped to derive by wintering in the south were much diminished.

We are glad to learn that an establishment will soon go into operation to which those suffering from such affections may resort with every confidence, both in regard to its hygienic management and the medical skill of its attending physician.

This *Sanitarium* for affections of the throat and lungs is to be under the care of Dr. N. D. Benedict, late Superintendent of the New York State Lunatic Asylum.

It is located at Magnolia, East Florida, on the river St. Johns, between Jacksonville and St. Augustine, at a distance of about twenty miles from the sea-board. It is one day's journey, by steam boat, from Savannah and Charleston, and four days, by steamer, from New York and Philadelphia, via Charleston or Savannah.

The climate of East Florida is probably better adapted, as a winter residence, for invalids with delicate lungs, than any other part of the

United States. The mean temperature of the winter months is about 60°; frost is rarely seen, and the little rain that falls is rapidly absorbed by a dry sandy soil, shaded by pine forests.

After much observation and deliberation, the above site was chosen, believing it to possess as many, if not more, advantages than other any location in that country.

The house, which is commodious, having large airy chambers, and in every respect well constructed for the purpose, will be opened in November next for the reception of invalids, who may or may not be accompanied by their friends.

We have known Dr. Benedict for many years, and can bear warm testimony, from personal experience, to his skill as a physician, and his warm-heartedness as a man. More particular information may be obtained by addressing him at Philadelphia.

---

We regret to state that there is no abatement in the ravages committed by the Yellow Fever in Virginia. The number of deaths in Norfolk average about 17 daily, and about 20 in Portsmouth. The papers inform us that Dr. Stone, of New Orleans, who has had an extensive acquaintance with the disease, besides being an accomplished physician, pronounces it to be of the same type as that which afflicted New Orleans in 1853, and to which the Creoles gave the name of *la peste.*

"It seems to differ from the old type of yellow fever in manner of attack, in treatment required, and in the celerity with which the work of death is performed—many of its victims dying within a few hours of their attack. The premonitory symptoms are simply a sharp acute pain like rheumatic pain, or sometimes a paralytic shock in some part of the body. For instance, Mr. Barclay was attacked by what felt to him like a severe blow upon the right hip. Others are attacked by a sharp pain across the knuckles of the hand. Unless attended to immediately, the pain extends up the arm, or leg, and gradually over the entire body. Internal fever ensues, while the skin and extremities are icy cold. The first effort is to bring about a reaction by wrapping the patient in ice, followed by hot mustard applications; the object being to produce perspiration, which, if successful, is generally followed by a cure. Very little, if any internal medicine is needed or allowed, except, perhaps, a slight tonic."

The Beacon, of Norfolk, has the following:

"Our panic-stricken citizens have fled and left nought behind them but pestilence and famine! They have carried off their spoils—their money—but they have left their city overwhelmed in present dismay and distress, with a long vista of adversity in prospective. Its present appearance is gloomy beyond conception. Take a position on Main, at the intersection of Bank street, and in ordinary times you command a view of the great business mart of the city, such as might convey by no means a faint idea of the New York "Broadway," on a small scale. But now how changed ! All the stores, with not more than half a dozen exceptions, closed on Main street and Market square, some score or two of persons passing, and these either members or employees of the Howard Association, on some errand of mercy. The rattling of wheels is heard as usual; but alas ! they are the wheels of physicians' carriages or of hacks used for despatch in conveying messengers. Go to any other part of the city and the same prevailing gloom is seen. The wharves are bare of shipping, and desolation reigns throughout their borders. All is cheerless and heart-sickening."

A letter writer under date of the 23d, also says :

"My estimate of 250 cases now under treatment, though formed on consultation with several of our leading physicians, seems to be too low. At the Dispensary alone 200 prescriptions a day have been put up for two or three days past, and there are five other apothecary establishments whose proprietors are as busy as they can be all day. Our doctors too, are going their rounds from early dawn to bed time. We know one who paid 68 visits in one day, another who had a list of 70 patients, another of 39, another of 31, another of 29, and we have about 15 of the faculty in good practice. You may form your own estimate from these figures. Drs. Peniston, Freeman and De Castro* are constantly on the go. The former paid 40 visits in Portsmouth yesterday, besides an almost equally large number here. All three gentlemen are doing noble service in the cause they have voluntarily espoused. Dr. Gooch, of Richmond, also came to this city yesterday, and paid a visit to the hospital at Julappi, where he made a careful inspection of the patients, every facility for that purpose being shown him by Dr. William M. Wilson, the accomplished and skilful resident. There are now 33 sick, and 47 convalescent in the hospital. The sick, for the most part, are doing very well, and it has been remarked, that in almost every case where the patient has been removed there when first attacked by the fever, he has rapidly recovered. The deaths have been few in comparison to the numbers sent, thus showing the virtue as well of pure air as of good attendance, neither of which the poor sufferers could enjoy if left at their homes in the city."

We are happy to say that the fever has sensibly abated in New Orleans during the last ten days.

